# 1756. Takeaways from Investigation of a Large Invasive Group A *Streptococcus* Outbreak in a Skilled Nursing Facility, Virginia, 2022-2023

**DOI:** 10.1093/ofid/ofad500.1587

**Published:** 2023-11-27

**Authors:** Rehab R Abdelfattah, Clarissa Bonnefond, Carolyn A Kiefer, Kayleigh Rehkopf, Holly E Spindle, Patricia Bair, Sarah Lineberger

**Affiliations:** Virginia Department of Health, Richmond, Virginia; Virginia Department of Health, Richmond, Virginia; Virginia Department of Health, Richmond, Virginia; Virginia Department of Health, Richmond, Virginia; Virginia Department of Health, Richmond, Virginia; Virginia Department of Health, Richmond, Virginia; Virginia Department of Health, Richmond, Virginia

## Abstract

**Background:**

Group A Streptococcus (GAS) primarily infects the respiratory tract, skin, and soft tissue. Invasive GAS (iGAS), in which the bacteria infect a normally sterile body site, is severe and can be life threatening, especially in older adults. The Virginia Department of Health investigated a prolonged iGAS outbreak in a skilled nursing facility from March 2022 - April 2023. Cases continued despite multipronged outbreak response.

**Figure:**
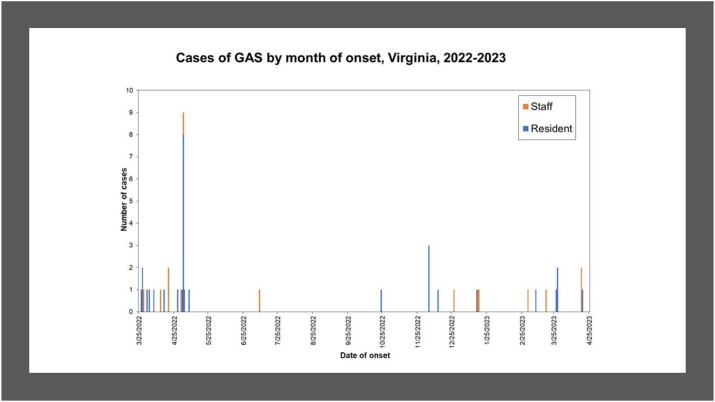

**Methods:**

A case was defined as GAS isolated from a sterile or nonsterile body site. Cases were identified through active surveillance, retrospective chart review, colonization screening by skin/throat swab, and culture. Multiple onsite visits were conducted to assess infection control practices at the facility. Genotyping (emm type) analysis was performed on resident and staff isolates.

**Results:**

From March 2022 to April 2023, 30 (71.4%) GAS cases were identified among facility residents, and 12 (28.6%) cases among staff. Ten residents (23.8%) died. GAS was isolated from throat (21, 50%), wounds (14, 33.3%), from more than one site (2, 4.8%), and invasive infections (5, 11.9%). Twenty (47.6%) cases were identified through colonization screening; the carriage rate among residents was 7% (16 positive out of 230) and 2.8% among staff (4 positives out of 142). Sequencing was conducted on 34 isolates, 33 (97%) were emm type 89 and were within < 10 single nucleotide polymorphisms (SNP) apart. Infection prevention assessments identified gaps in hand hygiene, wound care, and environmental cleaning and disinfection.

**Conclusion:**

Genotyping results suggested common source exposure. GAS might have transmitted several times between colonized or infected residents and staff which caused the extended outbreak. Multiple challenges at the facility contributed to a prolonged outbreak despite aggressive interventions. Challenges included high staff turnover; use of agency staff since the beginning of the COVID-19 pandemic; change in facility ownership; suboptimal infection prevention practices including wound care; and lack of appropriate active surveillance for GAS infections. Review of epidemiological data, frequent onsite infection prevention assessments, numerous educational sessions, and active follow-up were all crucial strategies for outbreak mitigation.

**Disclosures:**

**All Authors**: No reported disclosures

